# High-frequency and activation of CD4^+^CD25^+^ T cells maintain persistent immunotolerance induced by congenital ALV-J infection

**DOI:** 10.1186/s13567-021-00989-9

**Published:** 2021-09-15

**Authors:** Shuhai He, Gaoying Zheng, Defang Zhou, Li Huang, Jianguo Dong, Ziqiang Cheng

**Affiliations:** 1 College of Veterinary Medicine, Shandong Agricultural University, No 61, Daizong Street, Tai’an, 271018 Shandong China; 2 College of Husbandry and Veterinary, Xinyang Agriculture and Forestry University, No 1, North Ring Road, Xinyang, 464000 Henan China

**Keywords:** Avian leukosis virus subgroup J, CD4^+^CD25^+^ Tregs, viremia, immunotolerance

## Abstract

Congenital avian leukosis virus subgroup J (ALV-J) infection can induce persistent immunotolerance in chicken, however, the underlying mechanism remains unclear. Here, we demonstrate that congenital ALV-J infection induces the production of high-frequency and activated CD4^+^CD25^+^ Tregs that maintain persistent immunotolerance. A model of congenital infection by ALV-J was established in fertilized eggs, and hatched chicks showed persistent immunotolerance characterized by persistent viremia, immune organ dysplasia, severe imbalance of the ratio of CD4^+^/CD8^+^ T cells in blood and immune organs, and significant decrease in CD3^+^ T cells and Bu-1^+^ B cells in the spleen. Concurrently, the mRNA levels of IL-2, IL-10, and IFN-γ showed significant fluctuations in immune organs. Moreover, the frequency of CD4^+^CD25^+^ Tregs in blood and immune organs significantly increased, and the frequency of CD4^+^CD25^+^ Tregs was positively correlated with changes in ALV-J load in immune organs. Interestingly, CD4^+^CD25^+^ Tregs increased in the marginal zone of splenic nodules in ALV-J-infected chickens and dispersed to the germinal center. In addition, the proliferation and activation of B cells in splenic nodules was inhibited, and the number of IgM^+^ and IgG^+^ cells in the marginal zone significantly decreased. We further found that the mRNA levels of TGF- β and CTLA-4 in CD4^+^CD25^+^ Tregs of ALV-J-infected chickens significantly increased. Together, high-frequency and activated CD4^+^CD25^+^ Tregs inhibited B cells functions by expressing the inhibitory cytokine TGF-β and inhibitory surface receptor CTLA-4, thereby maintaining persistent immunotolerance in congenital ALV-J-infected chickens.

## Introduction

Avian leukosis virus subgroup J (ALV-J) is an oncogenic retrovirus identified in 1991. It is prevalent in all strains of chicken worldwide and cause tremendous economic losses to the poultry industry [1–3]. ALV-J induces persistent viremia or immunotolerance [4–6] before the emergence of numerous tumors. In general, the immunotolerance state is a prerequisite for tumorigenesis. During congenital infection of ALV-J, the cellular and humoral immune systems are severely damaged [7, 8], increasing the probability of chicken secondary infection by bacteria or viruses and mortality to a degree of magnitude higher than that caused by simple tumors induced by non-immunosuppressive viruses [9, 10].

A well-functioning mechanism of cellular and humoral immune response is necessary to resist persistent virus infection. Previous studies have shown that ALV-J infection cannot only cause damage to immune organs such as thymus and bursa of Fabricius in chickens, but also decrease the proliferative and killing activities of immunocytes [11, 12]. Our previous studies have revealed that B cell anergy caused by ALV-J infection is one of the important reasons for the immunotolerance induced by ALV-J [13]. However, whether the population and function change of T cells leads to B cells anergy during congenital ALV-J infection remains unclear.

Regulatory T cells (Tregs) comprise a mature T cell subset originating from the thymus with negative immune regulation function. Tregs play an extensive and powerful immunosuppressive role in peripheral immune responses, whether these are produced in the center immune organs or induced in the periphery. It has been confirmed that Tregs play an important role in retroviral infections [14]. Tregs are also closely related to the induction and maintenance of immunotolerance [15]. Many studies have shown that in some chronic infectious diseases, CD4^+^CD25^+^ Tregs inhibit the activation, proliferation, and secretion of cytokines from CD4^+^ and CD8^+^ T cells [16, 17] and also inhibit the proliferation and activation of B cells, thus mediating immunotolerance [18, 19]. Experimental data collected in human and animal have shown that the number of CD4^+^CD25^+^ Tregs in peripheral blood of subjects during virus chronic-infection is relatively higher than that of normal subjects [20–22]. Previous studies have shown that there are CD4^+^CD25^+^, CD4^+^CD8^+^CD25^+^, and CD8^+^CD25^+^ subsets in chicken, while CD4^+^CD25^+^ subsets have similar characteristics to mammalian regulatory T cells [23–25]. Research data show that chicken CD4^+^CD25^+^ Tregs play an immunosuppressive role by producing numerous pro-inflammatory cytokines and inhibiting the proliferation of immature T cells [26]. During viral infectious diseases, the involvement of CD4^+^CD25^+^ Tregs in immune regulation is one of the important reasons for chronic infection and immune escape of virus. Therefore, this study will focus on CD4^+^CD25^+^ Tregs to investigate the effect of CD4^+^CD25^+^ Tregs on B cells, and aim to clarify the possible role of CD4^+^CD25^+^ Tregs in ALV-J-induced immunotolerance.

## Materials and methods

### Materials

Fertilized eggs from specific-pathogen-free (SPF) white leghorns were purchased from the Saisi Company (Jinan, China). ALV-J (NX0101 strain [27]) and DF-1 cells were maintained in our laboratory. Chickens infected (*n* = 60) with ALV-J on embryo incubation day 6 (EID6) and control chickens (*n* = 60) were fed separately in isolated facilities. Other reagents/materials described in this article were purchased from commercial companies.

### Experimental design

The model of congenital ALV-J infection was established according to a previous experimental method [13]. The ALV-J infection group was prepared by injecting 100 μL 10^3.8^ TCID_50_ ALV-J suspension into the allantoic cavity on embryo incubation day 6 (EID6) and incubated at 37 °C with saturated humidity. Meanwhile, the control group eggs were incubated in separate incubators with the same incubation conditions. All experimental chickens were raised in rearing isolator under the same feeding conditions. At 7, 15, 30, 45, and 60 days post-hatching, the samples of blood, thymus, spleen, and bursa of Fabricius of the chickens were collected after the chickens were euthanized with pentobarbital sodium for the following experiments: (1) the p27 antigen of ALV-J in the cloaca and the titer of neutralizing antibody against ALV-J in blood were assessed by enzyme-linked immunosorbent (ELISA); (2) the effect of ALV-J on the morphology of immune organs was analyzed by gross dissection and microscopy; (3) the effect of ALV-J on CD3^+^, CD4^+^ T cells was analyzed by confocal laser scanning microscopy (CLSM); (4) the effect of ALV-J on CD4^+^, CD8^+^ T cells, and CD4^+^CD25^+^ Tregs in peripheral blood and immune organs of chickens was analyzed by fluorescence-activated cell sorting (FACS) analysis; (5) the viral load in chicken peripheral blood and immune organs was analyzed by absolute quantitative real-time PCR (q-PCR) analysis; and (6) the effect of ALV-J on the expression of cytokines in chicken immune organs and in CD4^+^CD25^+^ Tregs was analyzed by relative q-PCR.

Furthermore, to investigate the effect of ALV-J on the proliferation and activation capacity of B cells in the spleen germinal center (GC), chickens from the ALV-J-infected group and control group were injected with LPS (40 mg/kg) through the abdominal cavity 40 days post hatching. After five days, the spleens were collected and sectioned for immunohistochemical (IHC) detection.

### Gross and histopathological examination

The chickens were euthanatized and necropsied for the gross lesions and histopathology assays. Thymus, spleen, and bursas of Fabricius were collected from the chickens (ALV-J group, *n* = 10; control group, *n* = 10) and fixed in 4% paraformaldehyde. The tissues were processed for standard paraffin wax embedding; ~5 μm-thick sections were cut and stained with hematoxylin and eosin (H&E) for histopathological examination. Six random fields in each target tissue section were selected to determine the degree of pathological changes.

### ELISA

To assess viral shedding in chickens, cloaca swab samples were collected from the chickens (ALV-J group, *n* = 5; control group, *n* = 5) at 7, 15, 30, 45, and 60 days post hatching. The p27 antigen of ALV-J was detected using a commercial ALV-J antigen ELISA test kit (IDEXX USA Inc., Beijing, China) according to the manufacturer’s instruction. The levels of p27 antigen were evaluated by calculating the s/p ratio (cut-off: 0.2) as recommended by the manufacturer. Each biological sample was tested in triplicate.

### Neutralizing antibody titer assay

To detect ALV-J neutralizing antibody titer in ALV-J-infected chickens (*n* = 5), peripheral blood was obtained via venipuncture at 7, 30, and 60 days post-hatching. The sera from the peripheral blood were heat-inactivated at 56 °C for 30 min. The inactivated sera were serially diluted from 1:2 to 1:64. The NX0101 strain of ALV-J used in the neutralization assay were all titrated at 10^3.8^ TCID_50_. DF-1 cells were seeded into 96-well plates with 10^4^ cells per well. Approximately 100 μL of DMEM containing the virus were incubated with 100 μL serial diluted sera at 37 °C for 1 h and then transferred to the DF-1 cells cultured in 96-well plates. The medium was changed to DMEM with 1% fetal bovine serum and maintained at 37 °C and 5% CO_2_ for 5 days. At the end, DF-1 cells were freeze-thawed thrice and were tested for p27 antigen by ALV-J antigen ELISA test kit (IDEXX, Beijing, China) according to the manufacturer’s instruction.

### FACS analysis

At the age of 7, 15, 30, 45, 60 days, erythrocyte-depleted lymphocytes were obtained from blood, thymus, spleen, and bursa of Fabricius, and equal amounts of lymphocyte suspension from 3 chickens were pooled into one sample. Before analysis by flow cytometry for the proportions of CD3^+^, CD4^+^, CD8^+^ T cells, and CD4^+^CD25^+^ Treg subset, the lymphocytes were suspended in cold PBS and were labelled with Pacific Blue dye (Life Technologies) to detect dead cells. Meanwhile, these lymphocytes were stained with AF700-conjugated mouse anti chicken CD3 mAb, FITC-conjugated mouse anti chicken CD4 mAb, PE-conjugated mouse anti chicken CD8 mAb (Southern Biotech, Birmingham, AL, USA), or FITC-conjugated mouse anti chicken CD25 mAb (Bio-Rad, Hercules, CA, USA) for 30 min at 4 °C. In each round of analysis or sorting, use 2 mg/mL CD3 mAb, CD4 mAb, and CD8 mAb to label 1 × 10^6^ cells in 100 μL, and use 1 mg /mL CD25 mAb to label 1 × 10^6^ cells in 100 μL according to the manufacturer’s instruction. Lymphocytes were analyzed or sorted by a BD FACS Aria II instrument (BD Biosciences). In these tests, mouse IgG1κ-FITC and mouse IgG2a-PE isotype antibodies (Southern Biotech) were also used to establish the assay’s validity. The data were analyzed using FlowJo (TreeStar) software.

### Plasmid standard preparation

To analyze the viral load of ALV-J in the blood, thymus, spleen, and bursa of Fabricius of infected chickens, the recombinant plasmid containing the ALV-J *env* gene and *GAPDH* gene were constructed for establishing a standard curve. Conventional RT-PCR amplification of the *env* gene from ALV-J cDNA and *GAPDH* gene from DF-1 cell cDNA was conducted, and the primers used are listed in Table 1. Following amplification, the PCR fragments were cloned into the pMD-18T vector (TaKaRa, Shanghai, China) to obtain recombinant plasmids. The plasmids were extracted according to the instructions of OMIGA DNA plasmid purification kit (TaKaRa, Shanghai, China). The positive plasmids were tenfold (from 10^8^ to 10^1^ template copies per μL) serially diluted with ddH_2_O and used for the construction of the standard curve.

**Table 1:** **Primers for routine PCR amplification**

Purpose gene	Primer direction	Primer sequences (5′-3′)	Product length (bp)
*env* gene of ALV-J	Forward	TGCGTGCGTGGTTATTATTTC	144
	Reverse	AATGGTGAGGTCGCTGACTGT	
GAPDH gene of chicken	Forward	GAACATCATCCCAGCGTCCA	132
	Reverse	CGGCAGGTCAGGTCAACAAC	

### Quantitative real-time PCR analysis

To analyze the ALV-J load, mRNA levels of IL-2, IL-10, and IFN-γ in the thymus, spleen, and bursa of Fabricius, approximately 50 mg tissue were sampled for qPCR. To analyze the mRNA levels of TGF-β and CTLA-4 in the CD4^+^CD25^+^ Tregs, approximately 1 × 10^7^ CD4^+^CD25^+^ Tregs were sorted out for qPCR. Total RNA was extracted using TRIzol reagent (Invitrogen, Carlsbad, CA, USA) according to the manufacturer’s instructions and reverse transcribed to cDNA using the Taqman Gold Reverse Transcription kit (Applied Biosystems, Shanghai, China). Each 20-µL reaction contained 1 µL cDNA, 0.4 µL Rox Reference Dye II (50 ×), 10 µL SYBR Premix Ex Taq™ (TaKaRa, Shanghai, China), and 8 pM primers for ALV-J load as shown in Table 1 or 10 pM primers for IL-2, IL-10, IFN-γ, TGF-β, and CTLA-4 as shown in Table 2. For the assessment of mRNA relative expression levels of cytokines, the mRNA level of GAPDH was used as the internal reference. The reactions were run on a Light Cycler 96 Real-Time PCR machine (Roche, Basel, Switzerland) using the following program: 5 min at 95 °C; followed by 45 cycles of 95 °C for 5 s and 60 °C for 30 s to get a final melting curve. The mRNA levels of cytokines were analyzed using the 2^−ΔΔCt^ method, and ALV-J viral load was measured according to an absolute quantification equation: *y* =  −3.3519*x* + 38.532.

**Table 2:** **Primers for qRT-PCR amplification**

Purpose gene	Primer direction	Primer sequences (5′-3′)	Product length (bp)
IL-2	Forward	TTCATCTCGAGCTCTACACACCAA	200
	Reverse	GCATTCACTTCCGGTGTGATTTA	
IL-10	Forward	GAGCTGAGGGTGAAGTTTGAGGA	85
	Reverse	GTTCAGAGCTGAGCAGTTGGATGT	
IFN-γ	Forward	AGCATTTGAACTGAGCCATCACC	181
	Reverse	CCGTCAGCTACATCTGAATGACTTG	
TGF-β	Forward	ACCTCGACACCGACTACTGCTTC	126
	Reverse	CCATATAACCTTTGGGTTCGTGGA	
CTLA-4	Forward	TCTGCAAGATGGAGCGGATG	174
	Reverse	CGACAATGGCTGAGATGATGATG	

### Confocal laser scanning microscopy analysis

To examine the CD4^+^, CD25^+^, or CD3^+^ T cells, approximately 10-μm-thick frozen sections of spleen were prepared using the conventional method. Sections were fixed in ice-cold methanol for 3 min, and blocked with PBS containing 10% FBS for 10 min at room temperature. Then, these sections were incubated with PE-conjugated mouse anti-chicken CD4 monoclonal antibody (mAb; Southern Biotech, 1:500), FITC-conjugated mouse anti-chicken CD25 mAb (Bio-Rad, 1:500), or FITC-conjugated mouse anti-chicken CD3 mAb (Southern Biotech, 1:500) for 30 min at 37 °C. After staining cell nuclei with 4’,6-diamidino-2-phenylindole (DAPI), the sections were observed and analyzed with a SP8 CLSM (Leica, Wetzlar, Germany).

### Immunohistochemistry analysis

The chB6, peanut agglutinin (PNA), IgM, and IgG antigen in spleens were detected by IHC according to the instructions for the IHC test kits (ZSGB-Bio, Beijing, China). Briefly, after the antigen was retrieved and blocked with 10% nonimmune goat serum, tissue sections (consecutive sections were used for detection of chB6 and PNA) were incubated with the primary antibody at 4 °C for 12 h, and then the sections were washed with PBS thrice and incubated with the secondary antibody at 37 °C for 30 min. In this test, the primary antibodies included mouse anti-chicken chB6 mAb (1:200; Southern Biotech,), peanut agglutinin (PNA) mAb (1:1000; Bioss, Beijing China), rabbit anti-chicken IgM mAb (1:800; Abcam, MA, USA), and rabbit anti-chicken IgG mAb (1:800; Jackson, West Grove, PA, USA). The secondary antibodies included alkaline phosphatase-labelled goat anti-mouse IgG polymer and horseradish peroxidase-labelled goat anti-rabbit IgG polymer. Sections were stained with 5-bromo-4-chloro-3-indolyl-phosphate/nitro blue tetrazolium (BCIP/NBT), 3-amino-9-ethylcarbozole (AEC) or 3,3ʹ-diaminobenzidine (DAB) and counterstained with hematoxylin. Negative controls were also prepared using the same tissue.

### Statistical analysis

Two-way analysis of variance (ANOVA) was used to measure the difference significance of multiple sets of data. The unpaired *t*-test was used when two groups were compared. The results were accepted as significantly different when *p* ≤ 0.05, *p* ≤ 0.01, or *p* ≤ 0.001. Analysis and plotting of data were performed using Prism 8.0 (Graph Pad Software, San Diego, USA) and are expressed as the mean ± standard error of the mean (SEM).

## Results

### Congenital ALV-J infection causes immune organs stunting and immunotolerance

**Figure 1:**
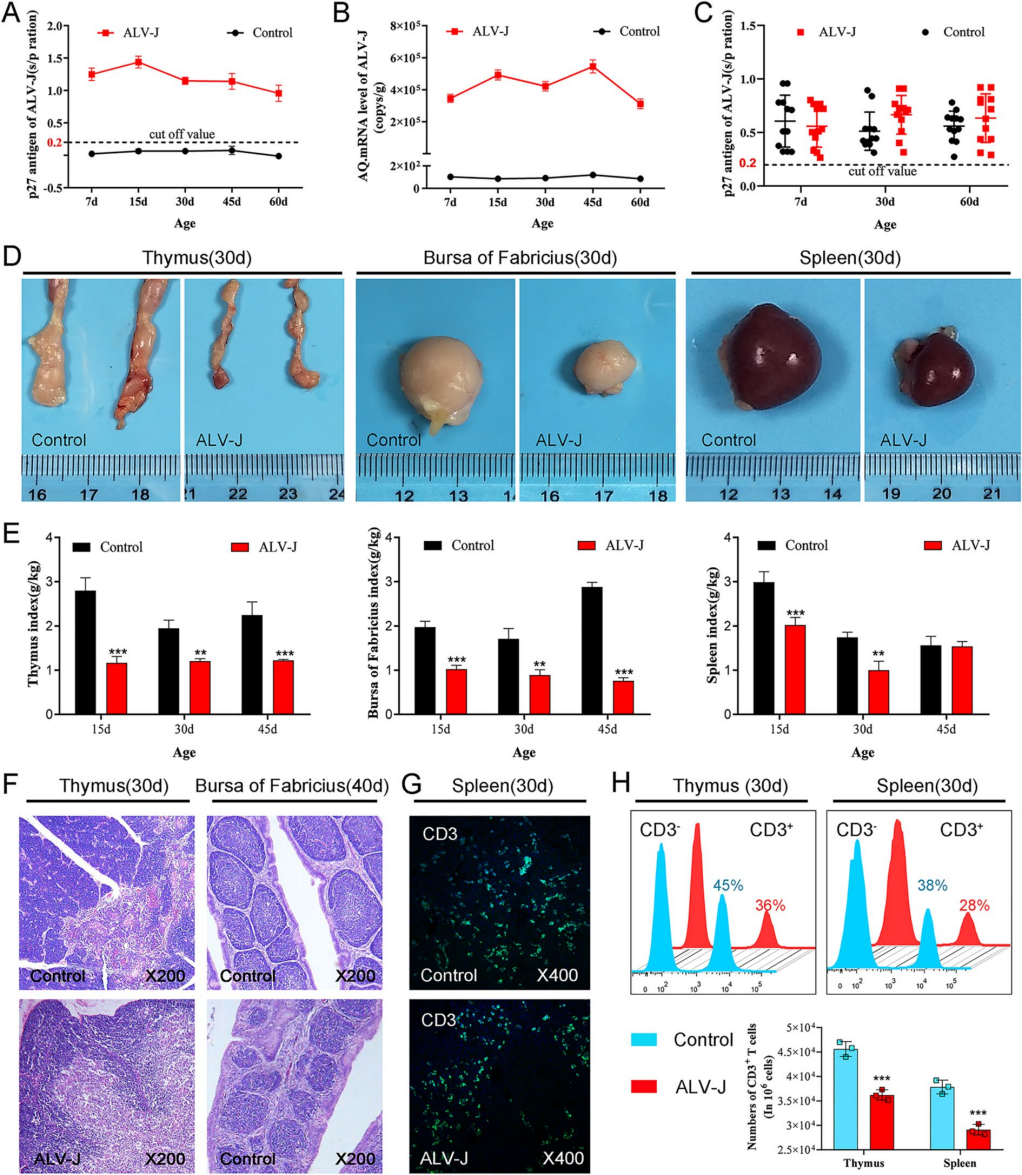
**Congenital ALV-J infection causes stunting of immune organs and immunotolerance.A** Detection of ALV-J antigen by ELISA in the cloacal swabs of chickens. **B** Detection of ALV-J antigen in peripheral blood by ELISA. **C** Anti-ALV-J neutralizing antibody titers in blood by ELISA. **D** Gross lesions of immune organs. **E** Dynamic changes in the immune index. **F** Histopathological detection of immune organs. **G** CLSM analysis of CD3^+^ T cells in the spleen. **H** FACS analysis of CD3^+^ T cells in the thymus and spleen. **p < 0.01, ***p < 0.001; the unpaired *t*-test was performed, and the data were expressed as mean ± SEM.

Cloacal swabs and blood samples were collected at 7, 15, 30, 45, and 60 days after hatching. The levels of p27 antigen and anti-ALV-J neutralizing antibody in ALV-J-infected chickens were detected by ELISA, and the ALV-J load in blood was assessed by q-PCR. Consistent with previous studies, chickens congenitally infected with ALV-J simulated by injection of yolk sac venom on the 6^th^ day of chicken embryo development developed severe immunotolerance, which showed continuous viral shedding (Figure 1A), persistent viremia (Figure 1B), and no neutralizing antibodies (Figure 1C). The results of gross autopsy further showed growth retardation in the thymus, bursa of Fabricius, and spleen from ALV-J-infected chickens (Figure 1D). The thymus and bursa of Fabricius indexes from ALV-J-infected chickens were significantly lower than those of the control group, and the spleen index was significantly lower than that of the control group before 45 days of age.

There was no significant difference in the spleen index between the ALV-J and control groups at 45 days of age, which may be related to the splenomegaly caused by ALV-J infection. Histopathological analysis further showed that the boundary between cortex and medulla of the thymus was not clear in ALV-J-infected chickens, the number of lymphocytes in medulla decreased but the number of dendritic cells increased (Figure 1F), the cortical area of bursa of Fabricius widened, medulla shrank, lymphoid follicles were dysplastic, and the number of lymphocytes decreased. CLSM and FASC analysis showed that the number of CD3^+^ T cells in the thymus and spleen significantly decreased after ALV-J infection (Figures 1G and H). These results suggest that ALV-J hinders the development and maturation of T cells and B cells in the immune organs of chickens.

### Abnormal proportion of CD4^+^ and CD8^+^ T cells in the blood and immune organs of ALV-J-infected chickens

**Figure 2:**
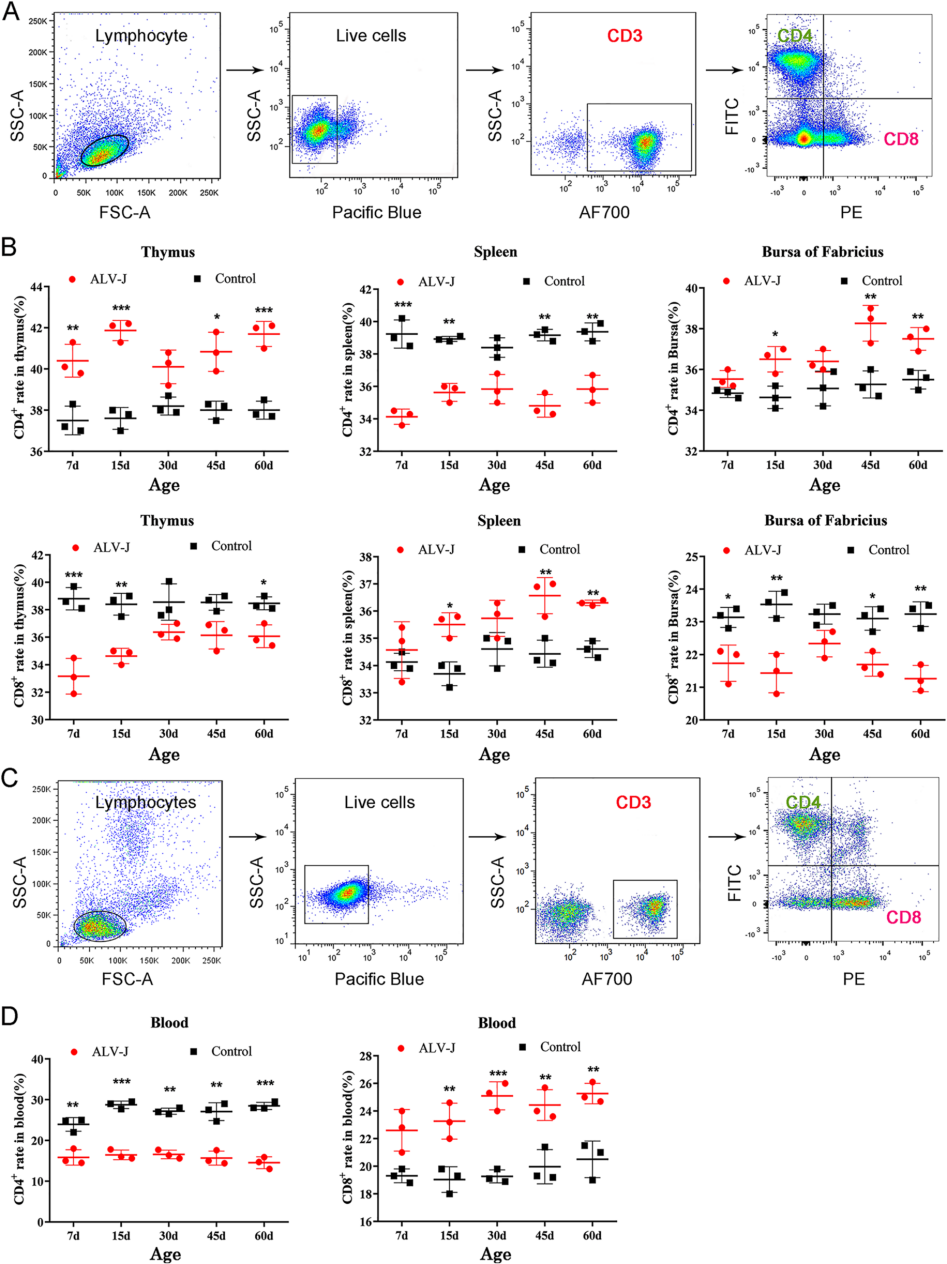
**The number and proportion of CD4**^**+**^**and CD8**^**+**^** T cells are abnormal in the blood and immune organs of ALV-J-infected chickens.A** FACS analysis strategy for CD4^+^ and CD8^+^ T cells in immune organs. **B** Quantitation of the frequency of CD4^+^ and CD8^+^ T cells in immune organs. **C** FACS analysis strategy for CD4^+^ and CD8^+^ T cells in blood. **D** Quantitation of the frequency of CD4^+^ and CD8^+^ T cells in blood. *p < 0.05, **p < 0.01, ***p < 0.001, the unpaired *t*-test was performed, and the data were expressed as the mean ± SEM.

To study the effect of ALV-J on T cells, the cell frequencies and dynamic changes of CD4^+^ T cells and CD8^+^ T cells from the peripheral blood and immune organs of chickens were analyzed by FACS (Figure 2A). As shown in Figure 2B, compared with the control group, the frequency of CD4^+^ T cells in the thymus and bursa of Fabricius of ALV-J-infected chickens significantly increased at 15, 45, and 60 days, but the frequency of CD4^+^ T cells in the blood and spleen significantly decreased. However, the frequency of CD8^+^ T cells in the above-mentioned tissues or organs was opposite to that of CD4^+^ T cells. These phenomena may be related to the antiviral response of the immune system. At the early stage of virus infection (7 days old), the frequency of CD4^+^ T cells in the thymus (the birthplace of T cells) was significantly higher than that in the control group, but the spleen and bursa of Fabricius showed a respectively significant decrease and no difference, which suggests that some special CD4^+^ T cell subset in the thymus had proliferated. These findings suggest that CD4^+^ T cells in vivo have proliferated, migrated, or redistributed after ALV-J infection, which leads to their imbalance in the proportion of immune organs and blood. Furthermore, the abnormal frequency and imbalance ratio of CD4^+^ and CD8^+^ T cells might be related to severe immunosuppression and persistent viremia in chickens infected with ALV-J. Another interesting phenomenon is that there was no significant difference in the frequency of CD4^+^ T cells or CD8^+^ T cells in the thymus, spleen, and bursa of Fabricius between the infected and control groups at the age of 30 days, which might be related to the relatively mature development of the immune system.

### ALV-J infection causes a decrease in cytokine IL-2 expression and increase in cytokine IL-10 expression in immune organs

**Figure 3:**
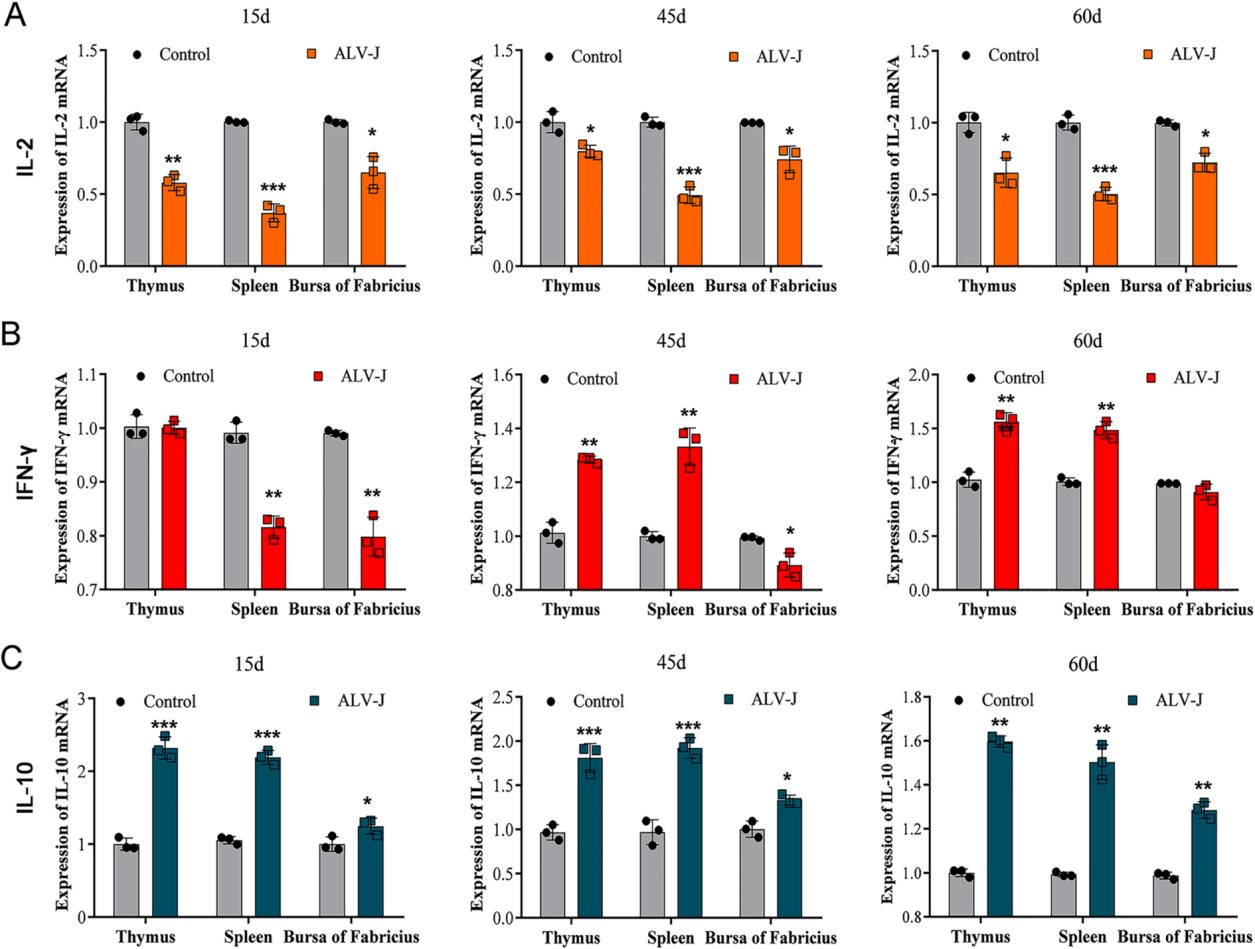
**ALV-J infection causes an increase in cytokine IL-2 expression and decreases in cytokine IL-10 expression in immune organs.A** RT-qPCR analyses of mRNA levels of cytokine IL-2 in immune organs of chickens at different time points. **B** RT-qPCR analyses for mRNA levels of cytokine IFN-γ in immune organs of chickens at different time points. **C** RT-qPCR analyses of mRNA levels of cytokine IL-10 in immune organs of chickens at different time points. *p < 0.05, **p < 0.01, ***p < 0.001, the unpaired *t*-test was performed, and the data were expressed as the mean ± SEM.

T cells play an important role in cellular immunity by secreting cytokines. IL-2 is mainly secreted by T helper cells (Th) Type 1, IFN-γ is mainly secreted by Th1 cells and CD8^+^ T cells, and these play a positive regulatory role. Moreover, IL-10 is mainly secreted by Th2 cells and Tregs cells, and these play a negative regulatory role. To further investigate the dynamic changes of cytokine secretion after ALV-J infection, the mRNA levels of IL-2, IFN-γ, and IL-10 in immune organs of 15-, 45-, and 60-day-old chickens were assessed by q-PCR. The results showed that the mRNA levels of IL-2 in immune organs from ALV-J-infected chickens significantly decreased (Figure 3A), but the mRNA levels of IL-10 significantly increased at 15, 45, and 60 days of age (Figure 3B). However, the mRNA levels of IFN-γ in the spleen and bursa of Fabricius of ALV-J-infected chickens significantly decreased at 15 days of age, but the mRNA levels of IFN-γ in the thymus and spleen significantly increased at 45 and 60 days of age (Figure 3C).

These results indicate that a T cell subset plays an inhibitory role in immune organs by secreting IL-10. The continuous decrease in cytokine IL-2 suggests that the function of CD4^+^ T cells such as Th1 cells was inhibited. However, the mRNA level of IFN- γ at different ages and in different immune organs was abnormal, which suggests an impairment of the immune function of ALV-J-infected chickens. These qPCR analysis results were confirmed by the results of FACS analysis of CD4^+^ T cells and CD8^+^ T cells (Figure 2B). FACS analysis showed that the number of CD4^+^ T cells significantly decreased, but the mRNA levels of IL-10 significantly increased, which indicated that the proportion of some immunosuppressive cell subset in CD4^+^ T cells increased, and the inhibitory effect was activated. At the same time, there is an interesting phenomenon that the frequency of CD4^+^ T cells in bursa of Fabricius significantly increased, but the mRNA levels of IL-2 and IFN-γ significantly decreased. This phenomenon indicated that the CD4^+^T cells in bursa of Fabricius contained some suppressive immune cell subset that can secrete negative regulatory cytokines.

### ALV-J infection causes an increase in the frequency of CD4^+^CD25^+^ Tregs in immune organs and blood

**Figure 4:**
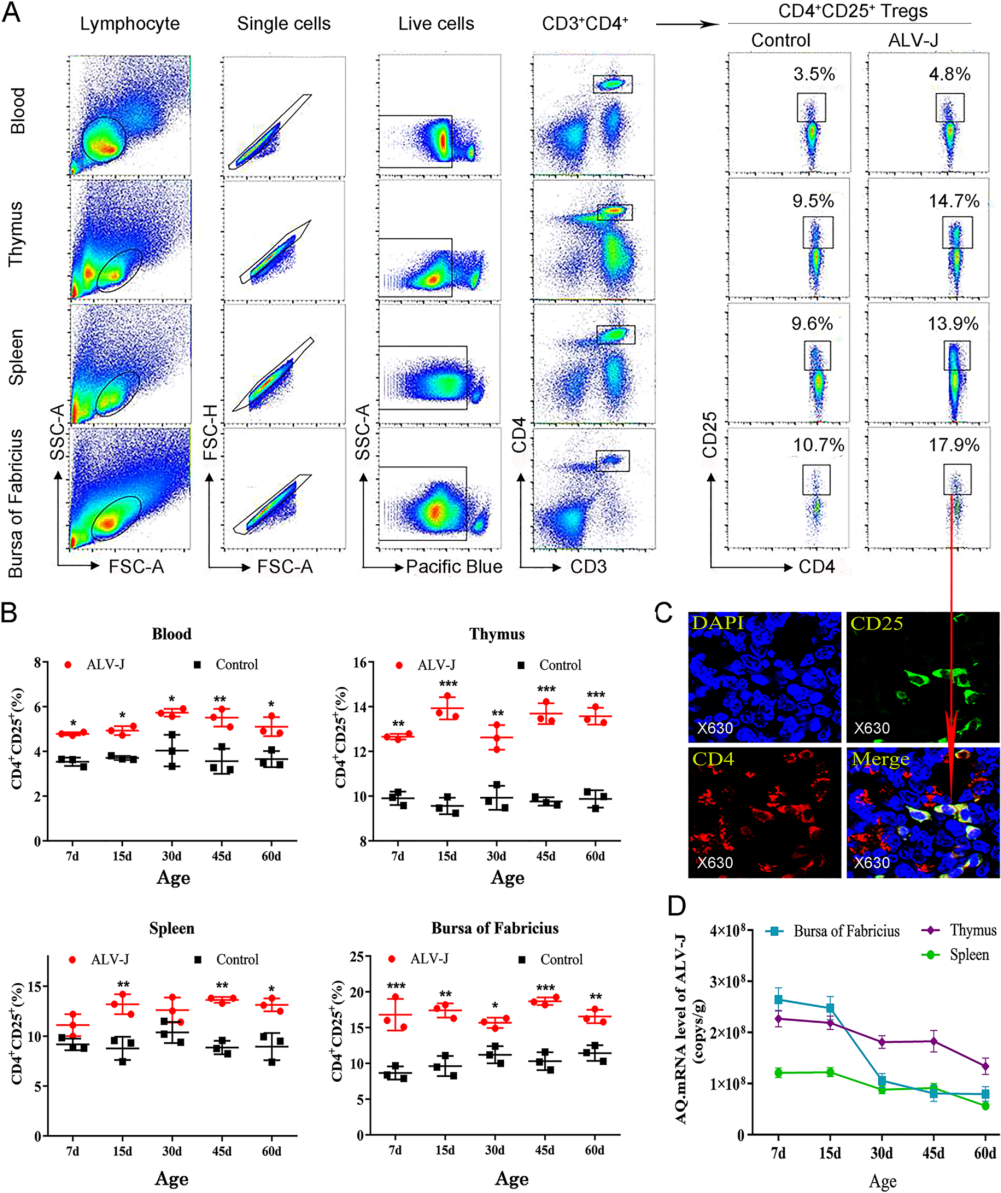
**Congenital ALV-J infection causes an increase in the frequency of CD4**^**+**^**CD25**^**+**^**Tregs and high viral load.A** FACS analysis strategy for CD4^+^CD25^+^ Tregs. **B** Quantitation of the frequency of CD4^+^CD25^+^ Tregs in blood and immune organs. **C** CLSM detection of CD4^+^CD25^+^ Tregs in the thymus of chicken. **D** ELISA results of viral load of ALV-J in immune organs. *p < 0.05, **p < 0.01, ***p < 0.001, the unpaired *t*-test was performed, and the data were expressed as the mean ± SEM.

To further investigate the cause of abnormal increase in suppressive cytokines in the immune organs and its relationship with immunotolerance induced by ALV-J, the distribution and number of CD4^+^CD25^+^ Tregs in tissues and organs were analyzed by CLSM and FACS (Figures 4A and C). CLSM confirmed the existence of CD4^+^CD25^+^ Tregs in chicken immune organs (Figure 4C). As shown in Figure 4B, compared with the control group chickens, the frequency of CD4^+^CD25^+^ Tregs in the thymus, bursa of Fabricius and peripheral blood significantly increased in ALV-J-infected chickens at 7, 15, 30, 45, and 60 days of age, and that of CD4^+^CD25^+^ Tregs in the spleen of ALV-J-infected chickens significantly increased at 45 and 60 days of age, but there was no significant difference at 7 and 30 days of age, which might be related to the migration process of CD4^+^CD25^+^ Tregs from central immune organs to peripheral immune organs. The frequency of CD4^+^CD25^+^ Tregs most significantly increased at the early stage of virus infection (about 15 days old) and later stage (about 60 days old), while at about 30 days old, the frequency of CD4^+^CD25^+^ Tregs in the immune organs was slightly lower than that of the other days.

Furthermore, the frequency of CD4^+^CD25^+^ Tregs in the thymus and peripheral blood showed a consistent trend, which suggested that CD4^+^CD25^+^ Tregs were continuously produced in the central immune organ (thymus) and were transported to the peripheral blood. However, the changes in the frequency of CD4^+^CD25^+^ Tregs in the spleen and bursa of Fabricius were not exactly similar to that in the blood and thymus, which suggested that the CD4^+^CD25^+^ Tregs in these organs might be redistributed during anti-viral infection by the immune system. Interestingly, when the frequency of CD4^+^CD25^+^ Tregs in immune organs was low, the viral load of ALV-J was relatively low, especially at about 30 days old. These results suggest that persistent viremia in ALV-J-infected chickens is closely related to the high frequency of CD4^+^CD25^+^ Tregs.

### The proliferation and differentiation of B cells are significantly inhibited in the spleen when CD4^+^CD25^+^ Tregs appear at high frequency

**Figure 5:**
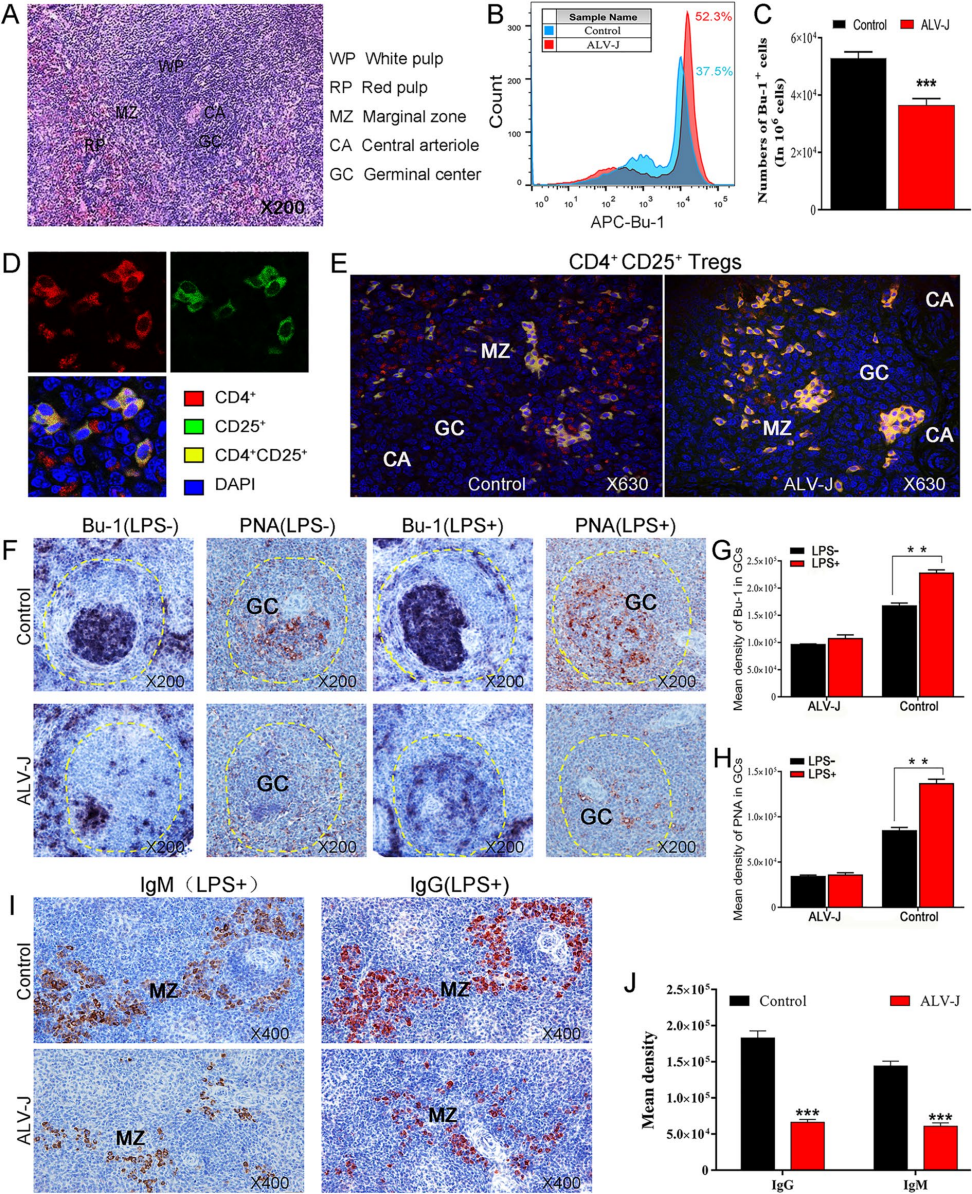
**CD4**^**+**^**CD25**^**+**^** Tregs disrupts the proliferation and differentiation of B cells after ALV-J infection.A** Histological structure of the chicken spleen. **B** FACS analyses of the proportion of B cells in the spleen of chickens. **C** Quantitation of **B**. **D**, **E** CLSM detection of CD4^+^CD25^+^ Tregs in the spleen of chickens. **I** IHC detection of B cells (Bu-1^+^ cells, black purple) and PNA^+^ cells (GC, brown). **G**, **H** Quantitation of **F**. **I** IHC detection of IgM^+^ plasma cells (brown) or and IgG^+^ plasma cells (red). **J** Quantitation of **I** **p < 0.01, ***p < 0.001, two-way ANOVA was performed, data were expressed as the mean ± SEM.

As mentioned above, congenital ALV-J infection causes severe immunotolerance in chickens, i.e., ALV-J-infected chickens cannot produce anti-virus neutralizing antibodies. Here, to further investigate the effect of high-frequency CD4^+^CD25^+^ Tregs on B cells, the relationship between the spatial distribution of CD4^+^CD25^+^ Tregs in immune organs and the proliferation and differentiation of B cells was studied. As shown in Figure 5A, the histological structure of chicken spleen includes the white pulp, marginal zone, and red pulp [28]. The lymphoid nodules in the white pulp are mainly composed of B cells that proliferate to form the germinal center (GC) under antigenic stimulation and the assistance of T cells, and most of the fully activated B cells differentiate into plasma cells that can secrete specific antibodies. The marginal zone is an important channel for lymphocytes to enter the spleen tissue, and it is also an important place for T and B cells to respond to antigen stimulation. FACS analysis showed that the number of B cells significantly decreased in the spleen of ALV-J-infected chickens (Figures 5B, C). CLSM analysis showed that CD4^+^CD25^+^ Tregs in the marginal zone of the spleen from ALV-J-infected chickens increased and dispersed in the germinal center (Figure 5E). These results suggest that CD4^+^CD25^+^ Tregs have an important effect on the biological properties of B cells during ALV-J infection. Furthermore, we examined the effects of ALV-J on the activation and proliferation of spleen B cells and GC formation. As shown in Figure 5F, after being stimulated by LPS, the B cells in the spleen corpuscle of the control group chickens proliferated to form GC (PNA-positive product) containing numerous B cells (Bu-1-positive product), and then, these activated B cells further differentiated into IgM^+^ and IgG^+^ plasma cells and migrated to the marginal zone and red pulp (Figure 5I). In sharp contrast, the B cells in the splenic corpuscles of ALV-J-infected chickens were not sensitive to antigen stimulation, and there was a major obstacle to the proliferation and activation of B cells, which was mainly characterized by the severe scarcity of Bu-1- and PNA-positive products in the splenic corpuscles. The number of IgM^+^ and IgG^+^ plasma cells in the marginal zone and red pulp was also significantly lower than those in the control group chickens. These results suggest that the CD4^+^CD25^+^ Tregs in ALV-J-infected chickens probably affect the proliferation and differentiation of B cells.

### Up-regulated expression of inhibitory cytokines and surface receptor in CD4^+^CD25^+^ Tregs after ALV-J infection

**Figure 6:**
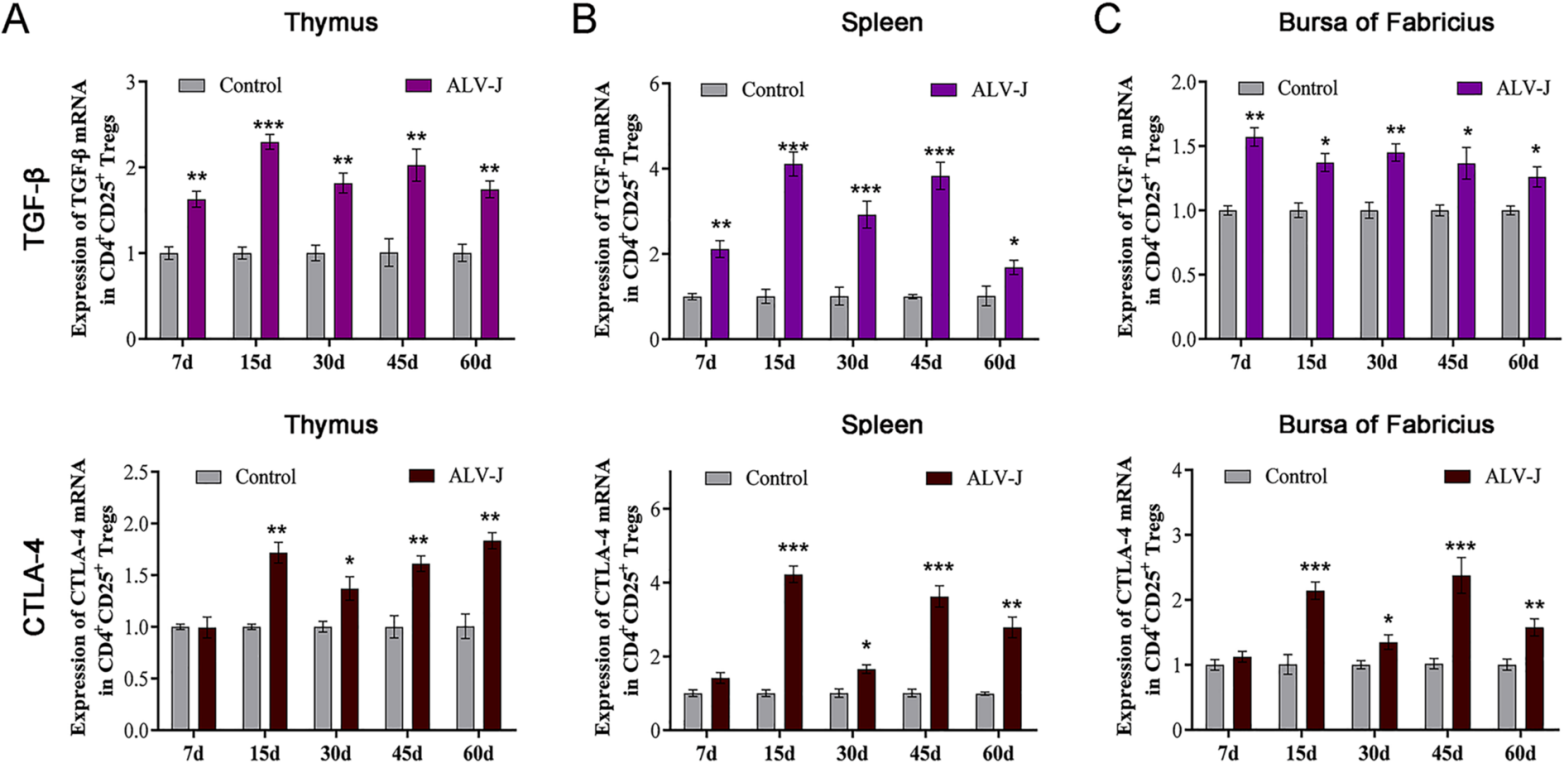
**Up-regulated expression of inhibitory cytokines and surface receptor in CD4**^**+**^**CD25**^**+**^** Tregs after ALV-J infection.A** RT-qPCR analyses of mRNA levels of TGF-β in CD4^+^CD25^+^ Tregs from immune organs. **B** RT-qPCR analyses of mRNA levels of CTLA-4 in CD4^+^CD25^+^ Tregs from immune organs. *p < 0.05, **p < 0.01, ***p < 0.001, the unpaired *t*-test was performed, and the data were expressed as the mean ± SEM.

In mammals, CD4^+^CD25^+^ Tregs impart their immunosuppressive effect mainly through contact inhibition and non-contact inhibition. It has been reported that the CD4^+^CD25^+^ Tregs in poultry also have immunosuppressive function by secreting cytokines such as TGF-β (non-contact inhibition) or up-regulated expression inhibitory surface receptor such as CTLA-4 (contact inhibition). To further investigate the relationship between immunotolerance induced by ALV-J infection and CD4^+^CD25^+^ Tregs, the mRNA levels of IL-10, TGF-β and CTLA-4 of CD4^+^CD25^+^ Tregs in immune organs were detected by q-PCR. The results showed that TGF-β mRNA levels of CD4^+^CD25^+^ Tregs in immune organs of 7- to 60-day-old ALV-J-infected chickens were significantly higher than those of the control group (Figure 6A). The CTLA-4 mRNA level of CD4^+^CD25^+^ Tregs in the immune organs of 15- to 60-day-old ALV-J-infected chickens was higher than the control group, whereas there was no significant change at 7 days old (Figure 6B). These results suggest that ALV-J infection causes the proliferation and activation of CD4^+^CD25^+^ Tregs in the central and peripheral immune organs, and CD4^+^CD25^+^ Tregs mediates the anergy of B cells by imparting non-contact inhibitory effects by secreting TGF-β as well as contact immunosuppressive effects by expressing CTLA-4 receptor.

## Discussion

Congenital viral infection is usually accompanied by severe immunotolerance such as the lack of effective antiviral antibodies in patients with congenital infection with HIV and HBV [29, 30]. The ALV virus (also known as AIDS of bird) is a member of the retrovirus family. Outbreaks and epidemics of the ALV-J have occurred in recent years [2, 3], and have resulted in severe immunotolerance in avian species. However, little is known about the immunotolerance mechanism induced by ALV-J. Unlike mammals, birds, as egg-laying animals, can be easily and quickly used as experimental animal models, which has a great advantage for us to study the mechanism of ALV-J-induced immunotolerance. Our previous study has shown that ALV-J infection can lead to B cell anergy [13]. Here, we further analyzed the causes of B cell anergy induced by ALV-J from the perspective of T cells. After all, the activation of B cells and the production of antiviral antibody is heavily dependent on the assistance of T cells [31].

The imbalance proportion of CD4^+^ T cells and CD8^+^ T cells in the blood and immune organs and the abnormal secretion of cytokines found in this study were considered to provide a crucial immune microenvironment for ALV-J-induced immunotolerance. It was found that naive T cells differentiate into effector T cells and further secrete cytokines such as IL-2 and IFN-γto facilitate rapid clearance of the virus at the early stage of the infection [32, 33]. In contrast, congenital viral infection causes the weakening or loss of the ability of T cells to secrete cytokines and proliferate, which will directly affect host antiviral protection [34, 35]. After detecting the T cell population in peripheral blood of patients with persistent HBV infection, researchers suggested that Tregs play an important role in immunotolerance maintenance [36–38]. The abnormal proliferation and activation of Tregs in patients with persistent HBV infection can disrupt T cell immune regulation, which is mainly reflected in the change of the proportion of CD4^+^ T and CD8^+^ T cells [39], the severe inhibition of CD4^+^ T cell response, and the downregulation of T cell immune function through increased secretion of IL-10 [40]. An interesting phenomenon in this study was that although the number of CD3^+^ T cells in the immune organs of ALV-J-infected chickens was less than that of control chickens, the frequency of CD4^+^ T cells in CD3^+^ T cell population significantly increased. In addition, the mRNA levels of IL-2, which is positive regulatory cytokine, significantly decreased, but the mRNA levels of IL-10 and TGF-β, which are negative regulatory cytokines, significantly increased. These results suggest that some subset that plays a negative regulatory role in the high frequency of CD4^+^ T cells population had proliferated and activated. Coincidentally, these cell subsets are exactly the CD4^+^CD25^+^ Tregs that were detected in this study.

Concordant with the results of a previous study [13], our results confirmed once again that ALV-J infection can significantly reduce the ability of B cells to proliferate, activate, and differentiate into plasma cells. Importantly, CD4^+^CD25^+^ Tregs seem to be strongly involved in this important event. Previous studies have suggested that CD4^+^CD25^+^ Tregs not only inhibit the proliferation and activation of effector T cells, but they also inhibit the proliferation and activation of B cells [41, 42]. Tregs can potentially migrate to B cell areas in secondary lymphoid tissues and suppress T cell-dependent B cell Ig response, and Tregs can also directly suppress B cell response without the need to first suppress Th cells [19, 43]. In our present study, the frequency of CD4^+^CD25^+^ Tregs increased in the marginal zone of splenic nodules in ALV-J-infected chickens and dispersed in the germinal center, but the number of IgM^+^ and IgG^+^ cells in marginal zone significantly decreased, which provides more direct evidence for Tregs inhibiting Ig gene switch recombination in B cells. Many studies have shown that Tregs participate in and promote the progression and maintenance of many chronic infectious diseases. The frequency of CD4^+^CD25^+^ Tregs in the peripheral blood of patients with chronic HIV infection who did not respond to HIV treatment is higher than that of responders [44, 45], and in untreated patients with HIV infection, the frequency of Tregs in peripheral blood is positively correlated to the DNA of HIV [46, 47]. A study showed that the percentage of CD4^+^CD25^+^ Tregs in chicken peripheral blood lymphocytes increased with an increase in humoral tolerance [48]. Moreover, John and his colleagues suggested that TGF-β^+^ T cells, a subset of Tregs, maintained the immunosuppressive state of chickens infected with Marek’s virus [26]. In this study, we continuously detected the frequency of CD4^+^CD25^+^ Tregs in total CD4^+^ T cells in the immune organs and peripheral blood of 7- to 60-day-old chickens. The results showed that the frequency of Tregs in the central immune organs (thymus and bursa of Fabricius) of ALV-J-infected chickens was higher than that of the control group, especially in the early stage of virus infection (15 days old). Furthermore, the frequency of Tregs in blood and the immune organs still fluctuated at a high level with increasing age. We also found that the frequency of CD4^+^CD25^+^ Tregs was positively correlated with the RNA level of ALV-J in the immune organs, just as the frequency of Tregs was positively correlated with the DNA level of HIV [49]. In this study, the viral load and Tregs frequency were significantly decreased in 30-day-old chickens infected with ALV-J, which was presumed to be related to the gradual development and maturation of chicken immune organs. Previous studies have shown that the maturation time of peripheral immune organs in chickens is about 4 to 5 weeks after hatching [50].

CD4^+^CD25^+^ Tregs impart immunosuppressive effects mainly through release of cytokines (non-contact inhibition) and expression of immunosuppressive molecules such as CD25, FOXP3, and CTLA-4 (contact dependence inhibition) [51, 52]. CTLA-4 is the receptor of B7 molecules on the surface of antigen-presenting cells, blocking the positive stimulation signal mediated by CD28 molecules, and ultimately inhibiting the activation and proliferation of T cells and B cells [53, 54]. In poultry, CTLA-4 is also involved in the immune response of CD4^+^CD25^+^ Tregs in the contact dependence inhibition pathway [55]. TGF-β and IL-10 are the main inhibitory cytokines secreted by CD4^+^CD25^+^ Tregs. Moreover, CD4^+^CD25^+^ Tregs could express CD73 and CD39, which ultimately inhibited the proliferation of Th1 cells and the production of TNF and IFN-γ [56–58]. In this study, we found that the expression of TGF-β and CTLA-4 of CD4^+^CD25^+^ Tregs in the immune organs of congenitally ALV-J-infected chickens significantly increased. Based on the results of these studies, we hypothesize that in the early stage of ALV-J infection, due to the immature development of immune organs, rapid replication of virus stimulates the proliferation and activation of CD4^+^CD25^+^ Tregs in the central and peripheral immune organs. Hence, we suggest that the over-proliferation and activation of CD4^+^CD25^+^ Tregs disrupt the immune balance and affect the function of effector T cells and the proliferation and activation of B cells, which are also an important cause of the persistent viremia and immunotolerance induced by ALV-J. Unfortunately, in this study, we have not performed the functional experiment verification in vitro to demonstrate the direct inhibitory effect of CD4^+^CD25^+^ Tregs on B cells, which is the next task of this study.

Based on the experimental data in this study, we suggest that CD4^+^CD25^+^ Tregs proliferate and activate after ALV-J infection, and migrated to peripheral immune organs, such as the spleen and bursa of Fabricius. Furthermore, these suppressive subsets probably cause B cells anergy by releasing cytokine TGF-β and expressing surface receptor CTLA-4, which eventually caused persistent viremia and severe immune tolerance.
